# Fabrication, characterization, and thermal property evaluation of silver nanofluids

**DOI:** 10.1186/1556-276X-9-645

**Published:** 2014-11-29

**Authors:** Monir Noroozi, Shahidan Radiman, Azmi Zakaria, Sepideh Soltaninejad

**Affiliations:** 1 School of Applied Physics, Faculty of Science and Technology, Universiti Kebangsaan Malaysia, 43600 UKM Bangi, Selangor, Malaysia; 2 Department of Physics, Faculty of Science, Universiti Putra Malaysia, 43400 UPM Serdang, Selangor, Malaysia

**Keywords:** Silver nanoparticles, Nanofluids, Microwave heating, Photopyroelectric, Thermal diffusivity

## Abstract

Silver nanoparticles were successfully prepared in two different solvents using a microwave heating technique, with various irradiation times. The silver nanoparticles were dispersed in polar liquids (distilled water and ethylene glycol) without any other reducing agent, in the presence of the stabilizer polyvinylpyrrolidone (PVP). The optical properties, thermal properties, and morphology of the synthesized silver particles were characterized using ultraviolet-visible spectroscopy, photopyroelectric technique, and transmission electron microscopy. It was found that for the both solvents, the effect of microwave irradiation was mainly on the particles distribution, rather than the size, which enabled to make stable and homogeneous silver nanofluids. The individual spherical nanostructure of self-assembled nanoparticles has been formed during microwave irradiation. Ethylene glycol solution, due to its special properties, such as high dielectric loss, high molecular weight, and high boiling point, can serve as a good solvent for microwave heating and is found to be a more suitable medium than the distilled water. A photopyroelectric technique was carried out to measure thermal diffusivity of the samples. The precision and accuracy of this technique was established by comparing the measured thermal diffusivity of the distilled water and ethylene glycol with values reported in the literature. The thermal diffusivity ratio of the silver nanofluids increased up to 1.15 and 1.25 for distilled water and ethylene glycol, respectively.

## Background

The potential of thermal properties of nanofluids, colloidal dispersion of nanomaterial in a base fluid, is a candidate for various uses, which range from biological and biomedical applications to the new class of heat transfer fluids [1]. The thermal conductivity of the nanofluids is the main thermal property that has been focused in the majority of the experimental studies such as hot wire technique and parallel plate geometry [2-4]. Recently, however, a few techniques have been developed to measure the thermal diffusivity of nanofluids [4-6]. High electrical and thermal conductivity of silver nanofluids, as well as their extraordinary optical properties and their thermo-optical properties, could lead to their use in a variety of applications in the biological and biomedical fields, for example, in photothermal therapies. The surface plasmon resonance of silver nanoparticles (Ag NPs) also makes these materials candidates for applications in electromagnetic hyperthermia therapies [7,8]. However, the particle shape, size, and size distribution are crucial factors in these applications [9]; different Ag NPs can display different optical and thermal properties [10,11], and it is therefore important that during the preparation process, these parameters can be controlled and adjusted.

Different methods have been used for the synthesis of Ag NPs, including hydrothermal [12], sol-gel [13], and electrochemical [14] techniques. The microwave (MW) irradiation method offers many advantages, including simple processes, a short reaction time, a rapid heating rate, and a high and uniform heat distribution that is produced by the dielectric heating [15]. In a MW device, heating is caused by the interaction of the permanent dipole moment in a molecule with high frequency (2.45 GHz) electromagnetic waves. In short, the polarity of a solvent plays a significant role in the reaction process, and the higher polarity value of the solvent, the more efficiently a solvent couples with the MW energy. Many factors affect the polarity of a solvent, such as the dielectric constant (*ϵ*’), dielectric loss (*ϵ*”), and tangent delta (tan *δ* = *ϵ*”/*ϵ*’). The dielectric loss is the most indicative factor. The MW radiation can heat a material through its dielectric loss, which converts the MW energy into heat energy. Heat is generated in this way, and this heat may affect the particle morphology and particle size, as well as the particles' physical properties. The stability and thermal properties of a Ag nanofluid can therefore be modified by choosing the correct solvent for the formation of the Ag NPs in the MW heating method [16]. However, no detailed studies that investigate the effects of the solvent properties on the formation and size of NPs and their thermal properties exist in the literature. Motivated by this, distilled water (DW) and ethylene glycol (EG) were selected as solvents to study the thermal diffusivity and dispersion of Ag nanofluids under MW irradiation as a function of time.

Here, we report our study of the formation and thermal properties of Ag nanofluids produced using two different solvents (DW and EG) and different MW irradiation times. DW and EG have different properties, and the influence of these different solvent properties on the particle size, size distribution, and thermal diffusivity of the Ag nanofluids were investigated. The optical properties and morphology of nanoparticles were characterized using ultraviolet-visible (UV-vis) spectroscopy and transmission electron microscopy (TEM). A photopyroelectric (PPE) technique, an accurate and non-destructive method [17-20], was chosen to investigate the effects on the thermal diffusivity of silver nanofluid.

## Methods

### Synthesis

AgNO_3_ (99.98%, Merck, Darmstadt, Germany) was used as the Ag precursor, polyvinylpyrrolidone (PVP: MW =29,000, Sigma Aldrich, St Louis, MO, USA) was used as a stabilizer for the fabrication of Ag NPs. In the first set of experiments, AgNO_3_ (0.3 g) and PVP (0.3 g) were each dissolved separately in 25 ml of DW and were stirred for 15 min. The transparent AgNO_3_ solution was then added to the surfactant solution. The resultant solution (W0) was colorless and was stirred for 10 min; after cooling to a room temperature, the solution was placed in a Panasonic microwave oven (NN-K574MF, 1100 W, 2.45 GHz) for four different times of 20, 40, 60, and 90 s (W1-W4); the heating was paused after each 20 s to prevent intense boiling of the solvents and aggregation of the Ag NPs. The same procedure was followed for the preparation of Ag NPs in the EG suspension at four different times 20, 40, 60, and 90 s (EG1-EG4).

### Characterization

The fabricated colloidal Ag NPs were characterized using UV-vis spectroscopy (UV-1650 PC, Shimadzu, Kyoto, Japan) in the range of 200 to 800 nm. Microscopic TEM observations of the Ag NPs (in a dry condition) were performed using a TEM (Hitachi H-7100 electron microscopy, Chiyoda, Tokyo, Japan), and the individual particle size and particle size distributions were determined using UTHSCSA ImageTool (version 3.0) software. The TEM samples were prepared by dispersing a few drops of Ag colloid on a carbon film supported by a copper grid. The morphology of the NP samples was also studied using a field emission scanning electron microscope (FE-SEM, S-4700, Hitachi, Tokyo, Japan) operating at 5.0 kV.

### Photopyroelectric technique setup

**Figure 1:**
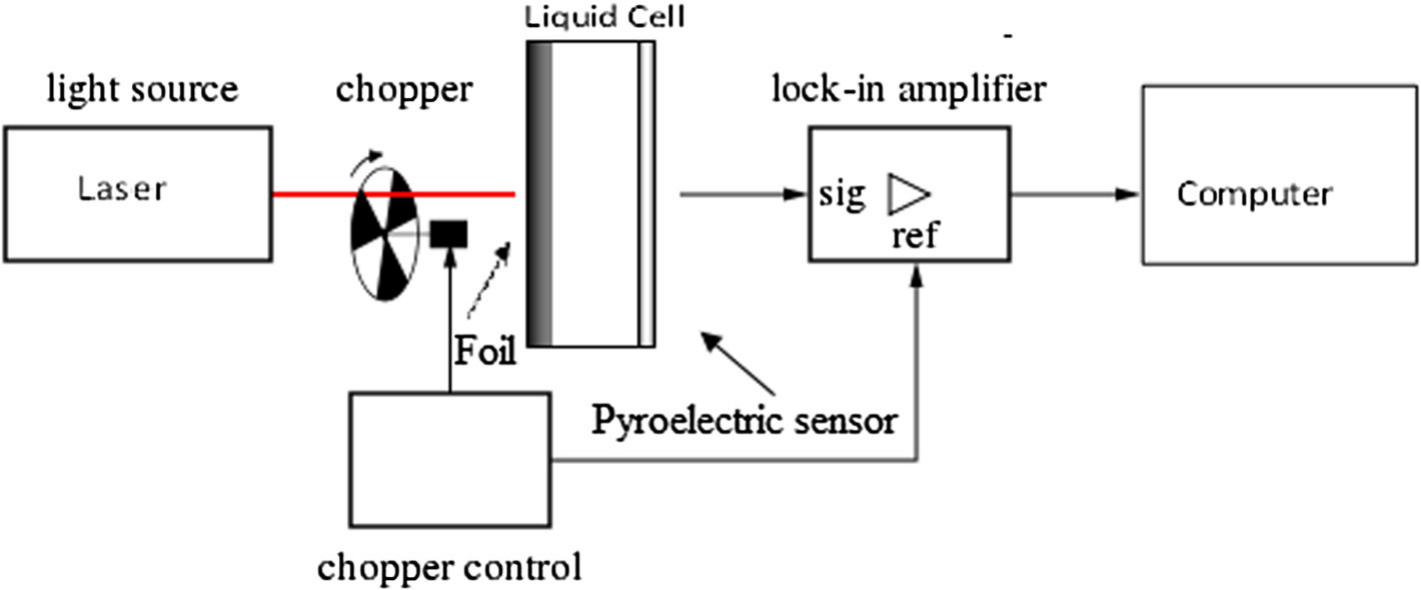
Schematic diagram of PPE technique.

The PPE setup can be found elsewhere [18]. It consist of a pyroelectric detector, a light source, data analyzing system, a lock-in amplifier, a metal light absorber (50-μm thickness), and a nanofluid cell. The modulated laser beam of a diode laser (532 nm, 200 mw) impinges on the black painted external face of metal foil was converted to thermal wave. The resulting thermal wave was transferred into the nanofluid sample. The pyroelectric (PE) detector, a polyvinylidene diflouride (PVDF: 52 micron, MSI DT1-028 K/L), is very sensitive to small changes in the heat flux; this PE detector was fixed with silicon glue to a Perspex substrate. The noise of signal was reduced by eliminating all the ground loops via proper grounding. The operating parameters were controlled through a computer equipped with adapted virtual instrument software, which allows automatic data acquisition (Figure 1). The PE signal was obtained by chopping frequency in 5 to 40 Hz and was performed at a room temperature (approximately 20°C). Before measuring the thermal diffusivity of the Ag nanofluids, a careful calibration of the experimental setup and procedures was performed, and the results were verified by measuring the thermal diffusivity of DW and EG (standard), prior to carrying out the actual measurements.

To describe the result of the experimental system, the temperature field can be calculated according to the thermal-wave cavity conduction-radiation theory [19]. The decay rate of PE signal *V* is determined by the thickness and thermal diffusivity of sample:

(1) $$V\left(f\right)=V_{0}\text{exp}\left(-\left(1+i\right)\sqrt{\frac{\pi f}{\alpha }}L\right)$$

(2) $$\ln\left|V\left(f\right)\right|=\ln\left|V_{0}\right|-\sqrt{\frac{\pi }{\alpha }}L\sqrt{f}$$

(3) $$\varphi =\varphi_{0}-\sqrt{\frac{\pi }{\alpha }}L\sqrt{f}$$

where *V(f)* is the complex PE signal, *V*_*o*_ and *φ* are the amplitude and phase of PE signal, *f* is the modulation frequency, *L* is the thickness, and *α* is the thermal diffusivity of sample. The thermal diffusion length of sample is *μ* = (*α/πf*)^1/2^. The frequency scan of the PE signal provides the direct and absolute thermal diffusivity measurement of sample [20]. By plotting the ln(amplitude) and phase as a function of *f*^½^, the thermal diffusivity values can be calculated from the slopes of linear part of the both plots:

(4) $$\alpha =\pi L^{2}/{\left(\frac{1n\left(\left|v\right|\right)}{\sqrt{f}}\right)}^{2},\alpha =\pi L^{2}/{\left(\frac{\varphi }{\sqrt{f}}\right)}^{2}$$

## Results and discussion

### Preparation of stable Ag nanofluids in different solvents

**Figure 2:**
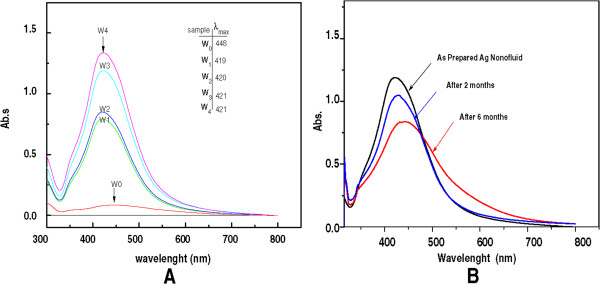
**UV absorption spectrum during formation of Ag + PVP nanofluids and absorption spectrum of W3 after storage. (A)** UV adsorption during the formation of Ag + PVP nanofluids (W_0_, W_1,_ W_2,_ W_3_. and W_4_ at before and after 20, 40, 60, and 90 s MW irradiation times) was on the traces. **(B)** Absorption spectrum of W3 after storage for 2 and 6 months at room temperature.

**Figure 3:**
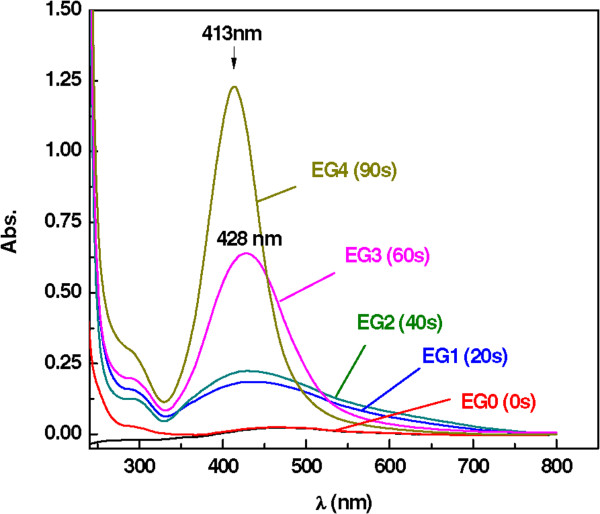
UV absorption spectra during the formation of Ag/EG nanofluids at the various MW irradiation times.

Figure 2 shows the absorption spectra of Ag/DW nanofluids under various MW irradiation times. The surface plasmon absorption band obtained of AgNO_3_/water suspension (W0), showed an absorption wide band at around 450 nm. The absorption peak intensity increased with the increment of MW irradiation time, 20 (W1), 40 (W2), 60 (W3), and 90 s (W4). The corresponding peak intensities of W1-W4 are in a range of wavelengths from 419 to 421 nm. The absorbance was directly proportional to the concentration of the NPs. Hence, the increasing absorbance with MW irradiation times, as shown in Figure 2, indicated that the total number of NPs in the solution had greatly increased [21]. The result showed the maximum absorption and a narrow size distribution of the Ag NPs achieved after 90 s of irradiation time, and this indicates that Ag nanofluid was highly homogeneous and the increase of absorbance is due to the increase the NPs concentration in the solution. To test the stability of the Ag nanofluid, the absorption spectra of sample W3 were measured after 2 and 6 months of storage at room temperature (Figure 2B). The absorption peaks were redshifted from 421 to 442 nm and they became broader. According to Mie theory, increases in the diameter of NPs result in a redshift in the absorption (Figure 2B) [22]; the results indicated that some agglomeration of the Ag NPs occurred in water over long time periods.Figure 3 shows the absorption spectra of the Ag/EG nanofluids under various MW irradiation times 0 s (EG0), 20 s (EG1), 40 s (EG2), 60 s (EG3), and 90 s (EG4) in the presence of PVP. Obvious changes in the peak position and the shape of the absorption spectra occurred with increases in the irradiation time. The absorbance for the range of wavelengths (200 to 800 nm) studied here was higher for the EG samples than the DG samples; the first peak appeared at approximately 300 nm, as in the water solution, but the second peak broadened at longer wavelengths from 443 to 428 nm for EG1 to EG3, respectively, and exhibited tailing effects that may have signified a variation in the size of the Ag NPs. Figure 3 also shows that for the solution irradiated for 90 s, the maximum peak intensity appeared at 413 nm, and the bands were sharper and more symmetrical, reflecting the more uniform size distribution and higher concentration of Ag.

The reaction process of Ag ^+^ reduction in EG can be represented by the equations (5) and (6) [15]. The uniform and rapid MW heating increases the reduction rate of Ag ions, which could facilitate the formation of NPs with a uniform particle distribution.

(5) $$CH_{2}OH‒CH_{2}OH\;\to \;CH_{3}CHO+H_{2}O$$

(6) $$2CH_{3}CHO+2Ag\left(I\right)\;\to 2Ag+2H^{+}+CH_{3}\mathrm{COCOC}H_{3}$$

The spectra obtained in Figure 3 with those in Figure 2 clearly show that the adsorption of EG was higher in comparison to the water solution. In both Figures 2 and 3, the peaks were sharp and symmetrical, signifying a narrow distribution in the size and morphology of the Ag NPs [23]. The high concentration and uniform distribution in the EG solution was attributed to the size of the Ag NPs. Some aggregation was observed, but the metallic Ag NPs prepared in EG were well dispersed and high in concentration, and the EG dispersions were much more stable than those prepared in water under identical MW irradiation conditions.

**Figure 4:**
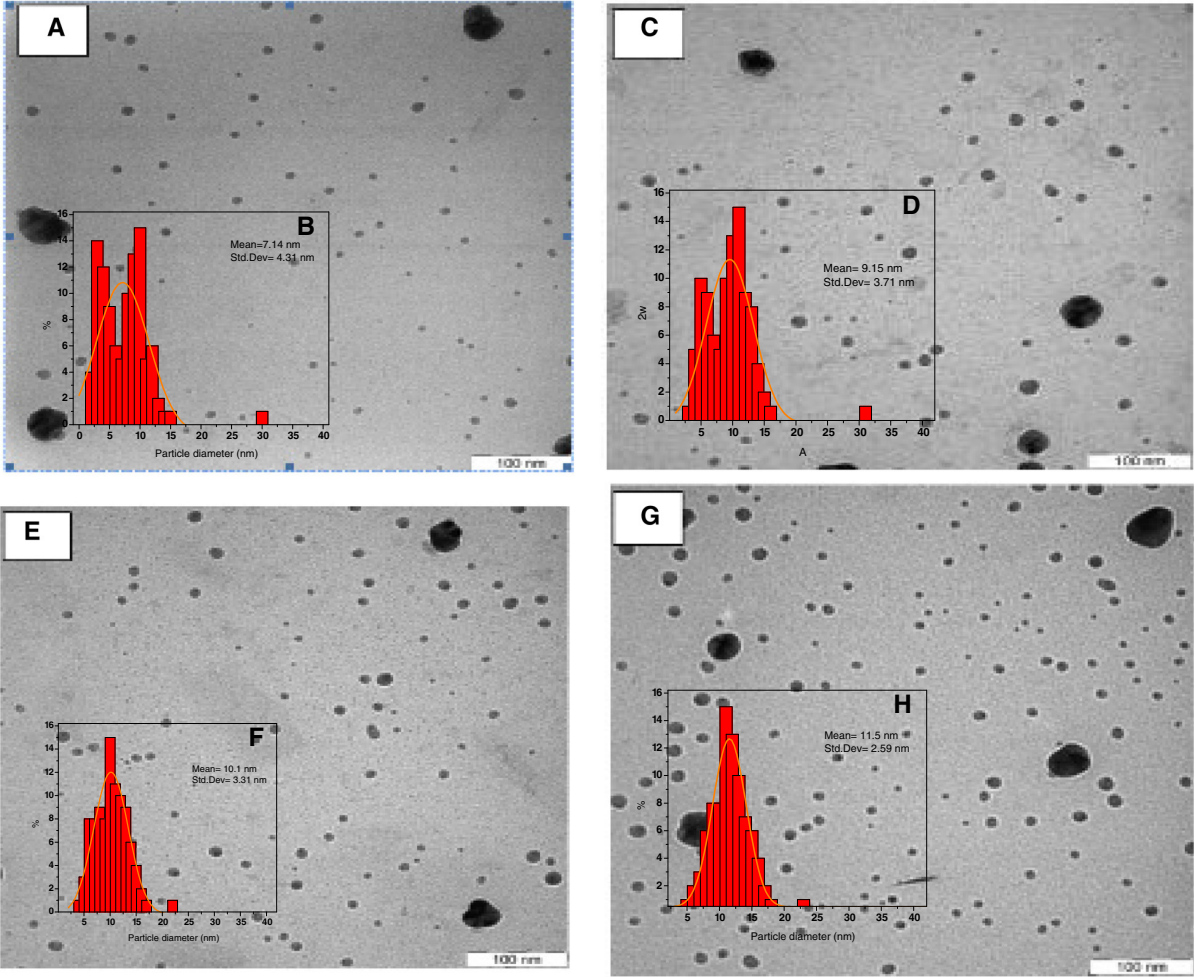
**TEM images and their size distributions of Ag NPs in DW at different MW irradiation time.** 20 **(A,B)**, 40 **(C,D)**, 60 **(E,F)**, and 90 s **(G,H)**.

Figure 4 shows TEM images, and corresponding size distributions, for Ag/DW nanofluids produced using various MW irradiation times. As shown in Figure 4, the mean diameter of the Ag NPs slightly increased with longer MW irradiation times and some aggregation was observed. When Ag nanofluids were prepared using irradiation applied for 20 and 40 s, the mean diameters were approximately 7.14 and 9.15 nm (Figure 4A,C), respectively. As seen in Figure 4E,G, when the irradiation time was increased to 60 and 90 s, the mean particle size increased significantly from 10.1 to 11.5 nm, respectively. However, there was an apparent increase in the NP concentration and, probably, an increase in the dispersion in the particle sizes after MW irradiation. This suggested that larger, and highly crystalline, Ag NPs were formed. However, the narrow size distribution and highly homogeneous nature of the Ag nanofluids prepared under 90 s of MW irradiation evidenced an increase in the concentration that was caused by an increase in the number of small NPs. The TEM image showed that the particle size distributions obtained the combined effect of PVP, which helped to control the particle size [15].

**Figure 5:**
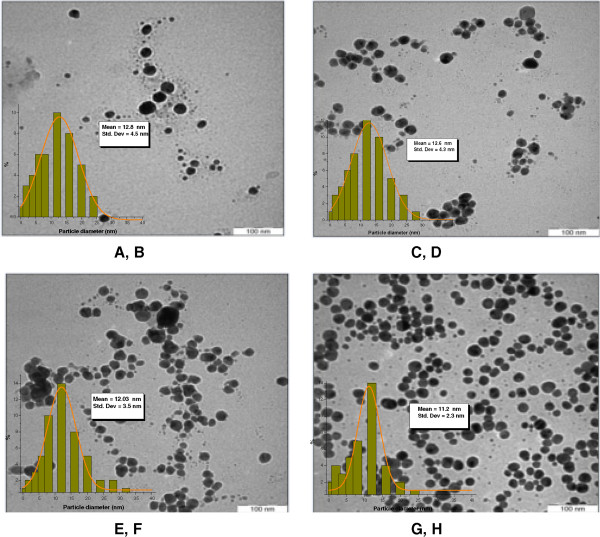
**TEM images and their size distributions of Ag NPs in EG at different MW irradiation time.** 20 **(A,B)**, 40 **(C,D)**, 60 **(E,F)**, and 90 s **(G,H)**.

Figure 5 shows TEM images, and corresponding size distributions, for the Ag/EG nanofluids produced using various MW irradiation times. In the Ag NP solutions prepared using 20 and 40 s of MW irradiation, Ag NPs were formed with a broad size distribution, and the mean particle size was approximately 12.8 and 12.6 nm, respectively (Figure 5A,C). For the solutions irradiated for 60 and 90 s (Figure 5E,G), the mean Ag NP particle size was decreased, but there was evidence of small species, as well as large. This was probably because the excess Ag^+^ ions produced more nuclei during the nucleation period, thus leading to the formation of smaller NPs [15]. More NP nuclei are required during the reaction process for the formation of small NPs with a narrow size distribution. The number and size of the small nuclei formed are controlled by the properties of the solvent. As shown by the results for the Ag/EG nanofluids, the special properties of EG, including its high-loss dielectric nature, high molecular weight, and high boiling point, resulted in increases in the total volume of the nanocrystals, and decreases in the size of the NPs, with time. It can be seen that larger Ag NPs were obtained under shorter irradiation time, because usually more crystal nuclei were provided under further irradiation [16]. The TEM result of decreasing in the particle size of Ag NPs dispersed in EG at 90 s is in accordance with the blueshift of its absorption peak as shown in Figure 3.

**Figure 6:**
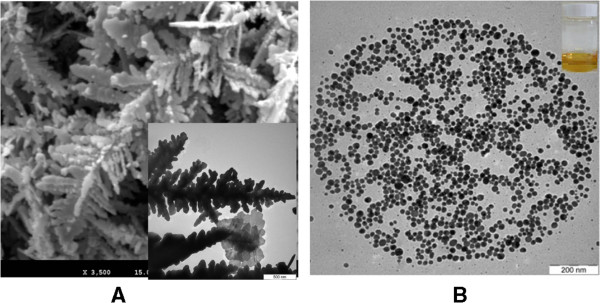
**TEM and FE-SEM images of the formation of dendritic and individual spherical nanostructures. (A)** TEM and FE-SEM image of the formation of dendritic nanostructures of Ag NPs in DW solution. **(B)** The formation of TEM image of the individual spherical nanostructure self-assembled of Ag NPs colloids in EG solution.

Figure 6(A) shows TEM and FE-SEM image formation of Ag NPs in DW irradiated at 90s MW irradiation time. As seen from Figure 6A, dendritic nanostructures resembling silver trees were formed when the Ag NP concentration was increased. The interaction between the Ag particles and the MW energy led to the growth of the NPs, and beautiful dendritic structures could be formed from the Ag NPs [24]. It was found that the concentration of AgNO_3_ played a significant role in the formation and growth of the dendrites, because the presence of a high number of closely located AgNO_3_ molecules in the solution led to the formation of the initial Ag NP aggregates. The initial Ag NP aggregates first arranged themselves in a linear shape, forming wire-like and rod-like structures, which became the beginnings of the dendritic structures [25]. These small structures grew in length and branched with other free NPs with increasing concentrations of AgNO_3_ in the solution. The TEM images in Figure 6(B) revealed a surprising effect. These images provided evidence of the spherical nanostructure of individual self-assembled Ag NPs with a diameter of approximately 1 micron, which were well-dispersed, and nearly monodispersed. The number of individual silver spherical nanostructures was large in the EG solution in which Ag NPs were formed under 90 s of MW irradiation (Figure 5G), and these structures tended to aggregate in one place [26,27]. The microwave expansion that occurs in the synthesis of nanocrystals is a rapid heating process that produces NPs that show very good monodispersity and crystallinity. However, MW irradiation should not be applied for too long (e.g., longer than 90 s), because the fast and efficient heating provided by MW irradiation is difficult to control.

### Comparison between Ag NPs in water and ethylene-glycol as a solvent

For both the DW and EG Ag nanofluids, it was shown that the use of PVP led to particles with a high degree of stability. Most of the Ag NP nanofluids prepared in water under MW irradiation were spherical and uniformly distributed; the small size and low concentration of the Ag NPs were illustrated by the TEM images shown in Figure 4. Figures 5 and 6 show that the Ag colloids obtained in EG under MW irradiation were nearly uniformly spherical in shape, well-dispersed, with a high Ag NP concentration, and a narrow size distribution. The differences between the Ag NPs formed in DW and EG were ascribed to the following factors:

1) Boiling point: The boiling point of the solvent has an important effect on the crystallinity of Ag NPs [28]. The high boiling point of EG (197.3°C) was advantageous for the high crystallinity of the Ag NPs and may have been the reason why EG was the favored solvent for the formation of Ag crystals under MW irradiation. The low boiling point of water did not favor the growth and ripening of crystals (Figure 4).

2) Molecular weight: In general, liquids with large molecules have high viscosity. In the case of EG, which has a large molecular weight, the long EG chains were easily adsorbed into the surface of the particles, owing to the higher viscosity of EG (1.61 × 10^−2^ Pa s) compared with that of water (8.94 × 10^−4^ Pa s) [29]; this assisted in the stabilization of the particles to achieve a narrow size distribution. This could also lead to the growth of crystals, and larger silver particles were also formed at high concentrations (Figures 5 and 6).

3) Dielectric loss: The higher dielectric loss of EG (49.950) compared with that of water (9.889) may have been the reason that the reaction proceeded at a much faster rate. The particles grew larger in EG as a result of the continuous nucleation of Ag.

As a result, for both solvents (DW and EG), changes in the MW irradiation time mainly affected the particles' stability, rather than their size. This was probably because longer irradiation times produced a large number of nuclei, leading to the formation of a narrow particle size distribution with a small average size.

### Enhancement of thermal diffusivity

**Figure 7:**
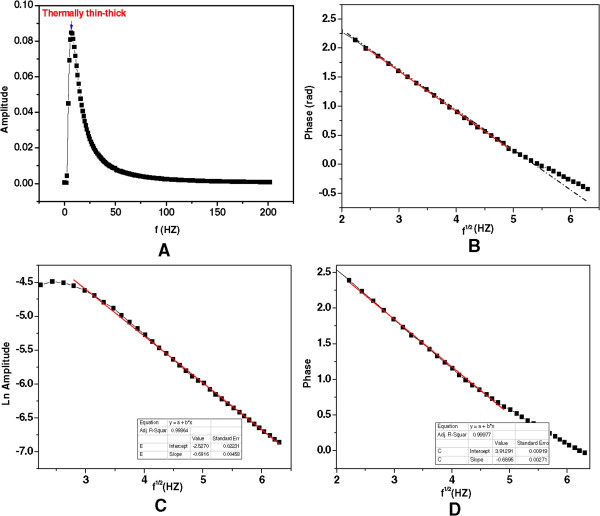
**The frequency behavior of the amplitude and phase of signal. (A)** The frequency behavior of the amplitude of signal obtained from DW, **(B)** ln amplitude of PE signal versus the square root of the frequency, **(C)** ln (amplitude), and **(D)** phase of PE signal versus the square root of the frequency for one of the samples.

**Figure 8:**
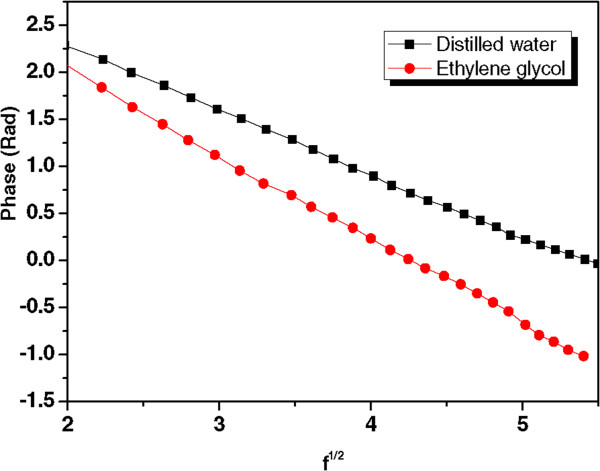
Variation of PE signal phase as function of square root of frequency for DW and EG solvents.

Figure 7A shows the PE signal of water, which was applied as a reference sample with known thermal properties, at modulated frequencies between 0.5 and 200 Hz. As shown in Figure 7A, at low chopping frequency, the thermal diffusion length was larger than the thickness of the sample, and the sample was in the thermally thin regime. When the frequency was increased, the sample became thermally thick, and the thermal waves penetrated into the sample; the detector then produced a PE signal, and this PE signal decreased exponentially with increasing frequency modulation. At high frequencies in the range of 50 to 200 Hz, the anomalous signal was very small and largely independent of the frequency [30]. The PE signals were therefore fitted only in the thermally thick area, and the curves were linear in the useful frequency range of 5 to 30 Hz, as shown in Figure 7B. The average thermal diffusivity values were calculated from the slope of the linear part of the logarithmic amplitude and phase of the PE signal curves, using equations (2) and (3). The values also confirmed by this method as well, Figure 7C, which shows an acquired PE signal, plotted as the square root of the frequency versus the logarithmic amplitude and phase of the PE signal.Before measuring thermal diffusivity of the Ag nanofluids, the PE setup and measuring procedures were tested using DW and EG as a reference and the base fluid, respectively; the plots in Figure 8 show the signal phase as a function of the square root of the frequency for the solvents used.

**Figure 9:**
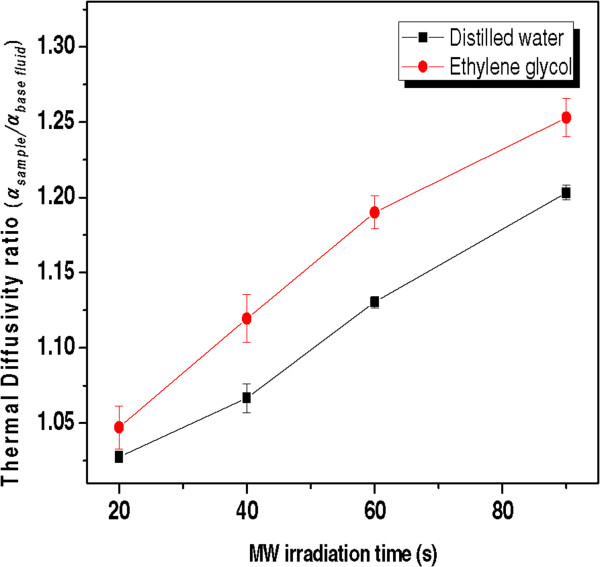
Thermal diffusivity ratio of Ag nanofluids with varying MW irradiation time for two solvents DW and EG.

The calculated average from PE amplitude and phase of water was 1.437 ± 0.030 × 10^−3^ cm^2^/s and for EG it was 0.925 ± 0.031 × 10^−3^ cm^2^/s; these values differed from literature values by less than 2% [31]. The thermal diffusivity ratios for these samples and other pertinent parameters are shown in Table 1 and are compared in Figure 9. The results showed that the thermal diffusivity of the Ag/DW and Ag/EG nanofluids was higher than that of either DW or EG [32].

**Table 1:** Summarized results for thermal diffusivity ratio of Ag nanofluids with varying MW irradiation time for two solvents DW and EG

	**Water**		**Ethylene glycol**	
**Time (s)**	**Thermal diffusivity(cm**^**2**^**/s) × 10**^**−3**^	**Thermal diffusivity ratio (α**_**sample**_**/α**_**base fluid**_**)**	**Thermal diffusivity** (**cm**^**2**^**/s) × 10**^**−3**^	**Thermal diffusivity ratio (α**_**sample**_**/α**_**base fluid**_**)**
20	1.476 ± 0.022	1.02	0.969 ± 0.009	1.04
40	1.532 ± 0.011	1.06	1.036 ± 0.011	1.12
60	1.624 ± 0.021	1.13	1.101 ± 0.006	1.19
90	1.729 ± 0.032	1.20	1.158 ± 0.007	1.25

Figure 9 shows the dependence of the thermal diffusivity ratio (*α*_sample_/*α*_base fluid_) for the Ag/EG and Ag/ DW nanofluids on the MW irradiation time. Here, when the MW irradiation time was increased, the total number of particles in the solution increased, and the thermal diffusivity ratio (*α*_sample_/*α*_base fluid_) of the nanofluids also increased, because of increases in the surface-to-volume ratio of the NPs, which led to decreases in the specific heat of the nanofluid [33]. Similar results have been reported in the literature for the thermal conductivity of Ag nanofluids, as measured using the transient hot-wires technique [34]. However, the enhancement of the thermal diffusivity ratio was smaller for the Ag/DW nanofluids than for the Ag/EG nanofluids. This dependence was attributed to the smaller particle size, the higher concentration, the uniform distribution, and the high surface area of the Ag NPs in the EG solution. These results confirmed that the Ag/EG nanofluids had good stability, and that they could be good candidates for bio-applications, including their use as heat-transfer nanofluids.

Previous studies have shown that the viscosity (not investigated here) and thermal properties of nanofluids increase when the concentration of NPs increases [35,36]. It is important to note that the methods used for the synthesis and preparation of nanofluids play a major role in improving the thermal properties of nanofluids, while the viscosity of nanofluids is an important transport property for applications in engineering systems. The ideal nanofluid should possess not only good thermal properties, but also low viscosity [37]. Maddah et al. showed that the viscosity increased slightly when the NP concentration was increased, and the enhancement in thermal diffusivity was larger than the enhancement in viscosity for Ag nanofluids [38]. Furthermore, Xie et al. studied ethylene glycol-based nanofluids containing five types of nanoparticles and discovered that nanofluids containing particles with an average diameter of less than 30 nm are appropriate for pumping in a heat exchanger setup [39]. The MW-assisted method therefore provides a promising route for the fabrication of NPs with small diameters, which result in monodispersed nanostructures, and which may in turn improve the thermal characteristics and decrease the viscosity of nanofluids.

## Conclusions

Stable and homogeneous Ag nanofluids were prepared in the distilled water and ethylene glycol, and the enhanced thermal diffusivity of these nanofluids was demonstrated. The synthesized Ag colloid was stable even after 6 months, and the average particle size in the different preparations was found to be between 7 and 12 nm. The results for the Ag NP dispersions indicated that the Ag ions interacted with the ethylene glycol much more strongly than with the water. This result, and the narrow size distribution, indicated that the higher dielectric loss, higher boiling point, and higher molecular weight of ethylene glycol (compared with water) may have had an important influence on the degree of crystallinity and growth of the Ag NPs. Ethylene glycol was a good medium for the synthesis, because of the high penetration depth of microwaves that it permitted. The thermal diffusivity of these nanofluids was investigated. The results showed that the thermal diffusivity was enhanced by the presence of the Ag NPs, compared with the results for the pure solvents. The thermal diffusivity ratio was found to increase with increases in the MW irradiation time; this led to increases in the nanoparticle concentration in the solvents. The thermal diffusivity ratio increased inversely with the thermal diffusivity of the nanofluid solvent. The enhancement of the thermal diffusivity ratio, when it was determined experimentally as a function of concentration for the Ag nanofluids, showed behavior similar to that observed in literature studies using different techniques. The PPE technique therefore provides a promising alternative method for the measurement of the thermal diffusivity of nanofluids with high accuracy.

## Competing interests

The authors declare that they have no competing interests.

## Authors’ contributions

MN carried out the experimental work, synthesis, characterization, and analysis and wrote the paper. SR and AZ supervised the experimental work and revised the manuscript. SS assisted in constructing the paper. All authors read and approved the final manuscript.
